# Chlorfenapyr-based Insecticide Induces Midgut Damage in the Tomato Leaf Miner *Phthorimaea absoluta* Meyrick, 1917 (Lepidoptera: Gelechiidae) Larvae

**DOI:** 10.1007/s13744-026-01376-9

**Published:** 2026-03-13

**Authors:** Filipe Schitini Salgado, Giovanna dos Santos Pereira, Laryssa Lemos da Silva, Renata Cordeiro dos Santos, Jéssica Letícia Abreu Martins, Jhersyka da Silva Paes, Marcelo Coutinho Picanço, José Eduardo Serrão

**Affiliations:** 1 Dept of General Biology, Federal Univ of Viçosa, Viçosa, Minas Gerais Brazil; 2 Dept of Entomology, Institute of Biotechnology Applied to Agriculture (BIOAGRO), Federal Univ of Viçosa, Viçosa, Minas Gerais Brazil

**Keywords:** Histochemistry, Insecticide resistance, Microbiota, Pirate®, *Tuta absoluta*

## Abstract

Pesticide resistance in agricultural pests has become a growing concern, as many species have developed resistance to most commercially available insecticides. *Phthorimaea absoluta* Meyrick, the tomato leaf miner, is one of the most destructive pests of tomato crops, capable of causing severe damage and even complete yield loss. This species has shown high levels of resistance to various insecticides, complicating effective pest management. Chlorfenapyr is a pro-insecticide that disrupts mitochondrial ATP production, ultimately leading to insect death. This study evaluated the lethal, sublethal, and histopathological effects of a chlorfenapyr-based insecticide on *P. absoluta* larvae through oral exposure. Lethal concentrations were determined using a concentration–mortality bioassay, and the LC_50_ value (3.72 ppm) was applied to assess histopathological alterations in the midgut. Chlorfenapyr-based insecticide exhibited high toxicity against *P. absoluta*. Histopathological and histochemical analyses revealed midgut epithelial damage, morphological alterations associated with cell degeneration, as well as the elimination of mycetocytes, which may play roles in digestion and xenobiotic detoxification. These findings provide one of the first histopathological characterizations of pesticide effects in this species and in microlepidoptera more broadly, reinforcing the potential use of chlorfenapyr within integrated pest management strategies.

## Introduction

One of the main challenges in agriculture is balancing productivity with environmental sustainability (Schoonhoven et al. 2005; Pedigo et al. 2021). The tomato, *Solanum lycopersicum* Linnaeus (Solanaceae), is an economically important crop and ranks among the most widely cultivated worldwide. A major obstacle to tomato production is infestation by the phytophagous insect *Phythorimaea absoluta* Meyrick (Lepidoptera: Gelechiidae), formerly *Tuta absoluta* (Meyrick, 1917) (Chang and Metz 2021). This leaf miner species has a global distribution and invasive potential across most continents (Santana et al. 2019), causing severe economic losses and even total crop failure (Haji et al. 1998). Due to the mining behavior of *P. absoluta* larvae, which penetrate plant tissues (leaf mesophyll, fruits, and stems), their exposure to contact insecticides is reduced, limiting the effectiveness of chemical control methods (Haji et al. 1998; Desneux et al. 2010). In addition, this species has developed resistance to some pesticides (Silva et al. 2016).

Insecticides typically act locally, targeting specific insect organs. They may cause damage at the point of contact, whether through the cuticle in topical applications or in the midgut when ingested (Denecke et al. 2018). As the primary site of pesticide absorption, the midgut plays a central role in toxicological studies. Histopathological evaluations are therefore essential to assess the cytological, histological, and systemic damage caused by insecticides (Fiaz et al. 2018; Dutra et al. 2019; Santos Junior et al. 2020).

The larval midgut of *P. absoluta* shares several morphological traits with other Lepidoptera, including a peritrophic matrix and a monolayered epithelium composed of digestive, goblet, and regenerative cells (Dos Santos et al. 2015; Fiaz et al. 2018). However, unlike other species in this order, *P. absoluta* has been reported to possess mycetocytes (Dos Santos et al. 2015), structures associated with hosting symbiotic microorganisms that contribute to digestion and may play a role in resistance to xenobiotics (Siddiqui et al. 2022).

Chlorfenapyr, a halogenated pyrrole pro-insecticide, is metabolized in insect cells by mitochondrial oxidases into its active form, which disrupts oxidative phosphorylation by inactivating components of the electron transport chain, ultimately inhibiting ATP production (IRAC 2017; Huang et al. 2023). This molecule has been reported as effective in controlling *P. absoluta* in tomato crops based on concentration–mortality studies (Gontijo et al. 2013; Hanafy & El-Sayed 2013; Silva et al. 2016). However, no data are available on its histopathological effects or potential sublethal damage in this species.

Most studies focus on insect mortality, whereas sublethal effects on internal organs remain largely overlooked. Such effects, particularly structural damage to the midgut, can severely compromise insect fitness (Schoonhoven et al. 2005; Desneux et al. 2007; Castro et al. 2020; Carneiro et al. 2020; da Silva et al. 2020; Farder-Gomes et al. 2021a, 2021b; Serra et al. 2021).

The aim of this study was therefore to assess the mortality and midgut histopathology of third-instar *P. absoluta* larvae exposed to a chlorfenapyr-based insecticide.

## Materials and methods

### Insects

Third-instar larvae of *P. absoluta* were obtained from colonies maintained at the Integrated Pest Management Laboratory of the Federal University of Viçosa, Viçosa, Minas Gerais, Brazil (20°45′N; 42°52′W). Colonies were reared in cages at 25 ± 1°C, 12-h photophase, and 75 ± 5% relative humidity and fed on tomato leaves (*Solanum lycopersicum*) (Galdino et al. 2011). Experimental insects were promptly submitted to treatments to minimize external and temporal interference.

### Concentration–mortality bioassay

The effects of the chlorfenapyr formulation Pirate® (240 g L⁻^1^ active ingredient and 880 g L⁻^1^ inert ingredients; BASF) were determined by estimating lethal concentrations (LC_25_, LC_50_, LC_75_, and LC_90_) under laboratory conditions. Five insecticide dilutions were prepared in 1 mL of distilled water: 1.2, 2.4, 6, 12, and 18 ppm, corresponding to 1%, 2%, 5%, 10%, and 15% of the maximum recommended field dose (MAPA 2022), as established in a previous experiment. Each treatment was obtained by diluting aliquots of a stock solution in distilled water. The five concentrations and a control (distilled water only) were applied in 1 mL aliquots using a micropipette to 0.25 g of tomato leaflets, which were allowed to dry at room temperature for 20 min before the larvae were introduced. The leaflets remained available to the larvae until the end of the experiment (Galdino et al. 2011) The bioassay was performed in 100 cm^3^ plastic cages with perforated plastic lids and a moistened cotton ball to maintain humidity. Each cage contained 10 *P. absoluta* larvae, with four replicates per treatment, totaling 40 individuals per treatment (*n* = 240). Mortality was evaluated after 48 h (Galdino et al. 2011).

### Histopathology

After the concentration–mortality bioassay, another set of larvae were exposed to the estimated LC_50_ of the chlorfenapyr-based insecticide for 48 h, and 10 live larvae (treated and control) were transferred to Zambonis’ fixative solution (Stefanini et al. 1967) for 24 h. Then the samples were dehydrated in a graded ethanol series (70%, 80%, 90%, and 95%) and embedded in Leica historesin (Leica Biosystems Nussloch GmbH, Heidelberg, Germany) following the manufacturer’s instructions. The larvae were sectioned at a three µm thickness using an RM2255 rotary microtome (Leica Microsystems, Wetzlar, Germany). Sections were stained with hematoxylin and eosin and examined under a Leica DMLS light microscope (Leica Microsystems GmbH, Wetzlar, Germany).

### Histochemistry

Sections from 10 control and 10 chlorfenapyr-exposed larvae, obtained from the same bioassay described above, were submitted to histochemical tests. Periodic Acid–Schiff (PAS) to detect polysaccharides and glycoconjugates, and mercury bromophenol blue for protein detection (Bancroft & Gamble 2008). This approach was chosen to ensure consistency and eliminate the risk of population-level variability between histopathological and histochemical analyses.

### Statistical analysis

Lethal concentrations (LC_25_, LC_50_, LC_75_, and LC_90_) and their respective confidence intervals and statistical parameters were estimated using Probit analysis based on the concentration–mortality model. Analyses were performed in R software for Windows (R Core Team 2024).

## Results

### Concentration-mortality bioassay

The concentration-mortality model provided a good fit to the data, allowing for the determination of toxicological endpoints, supporting the toxicity of the chlorfenapyr-based insecticide to *P. absoluta* third-instar larvae after 48 h (Table 1). Mortality was < 2% in the control.

**Table 1:** Lethal concentrations of chlorfenapyr-based insecticide for third-instar larvae of *Phthorimaea absoluta* 48 h after oral exposure, obtained by Probit analysis (d.f. = 3 slope ± sd = 2.2509 ± 0.4299 intercept = 5.4685 ± 1.0194)

Lethal concentration	Estimated concentration (ppm)	95% confidence limits (ppm)	χ^2^ (*p)*
LC_25_	1.87	0.36–3.33	7.56 (*p* = 0.0561)
LC_50_	3.72	1.61–6.75	
LC_75_	7.42	4.28–22.56	
LC_90_	13.80	7.42–93.38	

### Histopathology and Histochemistry

**Fig. 1:**
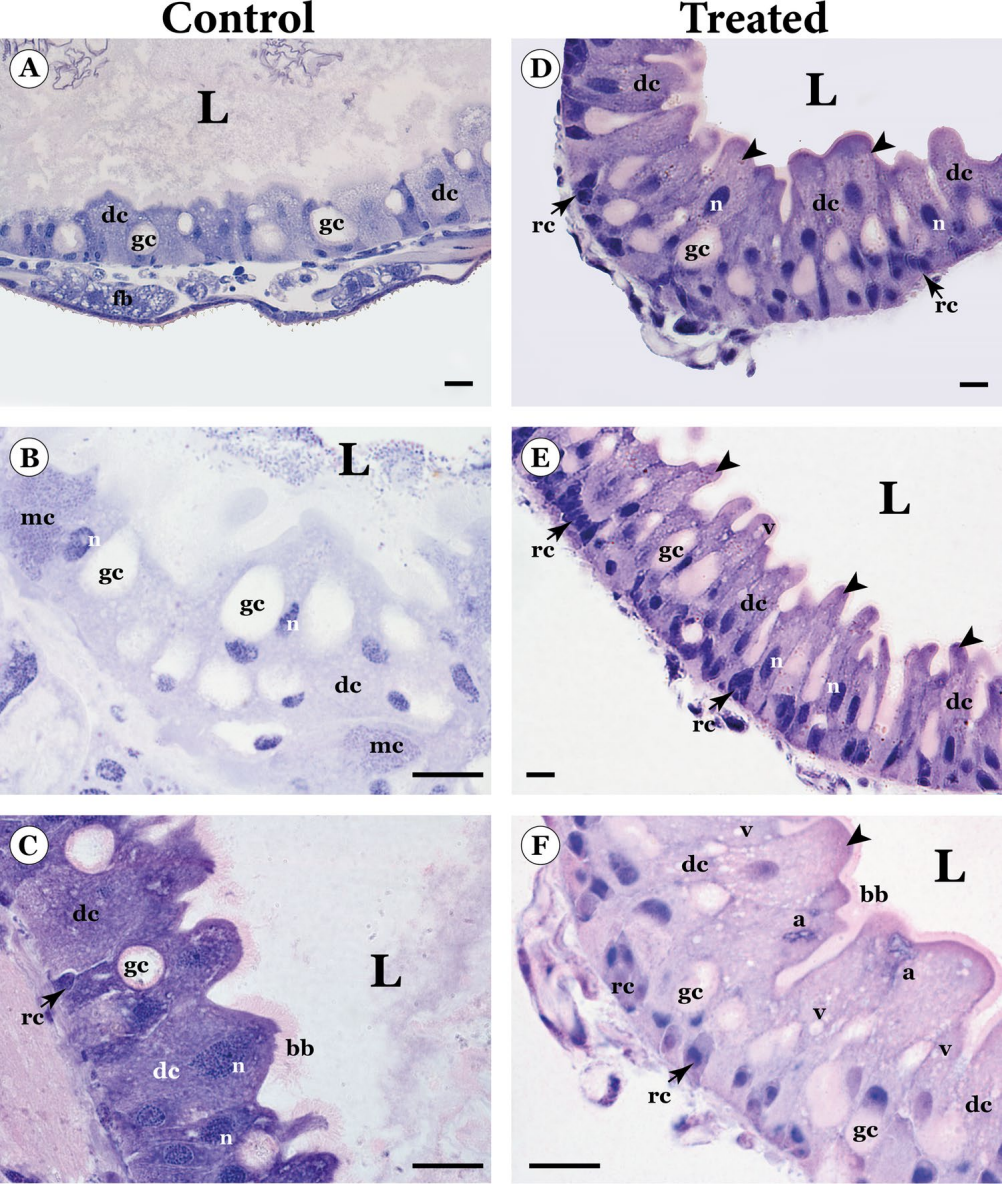
Light micrographs of the midgut of the third-instar larvae of *Phthorimaea absoluta* control and orally exposed (treated) to the chlorfenapyr-based insecticide. **A** General aspect of the midgut epithelium showing the digestive (dc) and goblet cells (gc). **B** Midgut epithelium showing mycetocytes (mc). **C** Midgut epithelium showing well-developed apical brush border (bb) and regenerative cell nest (rc). **D** Midgut epithelium showing regenerative cell nest (rc) and digestive cells (dc) with apical protrusions (arrowhead). **E** Disorganized midgut epithelium apical protrusions (arrowheads) and disorganized brush border (bb). **F** Midgut epithelium showing digestive cells (dc) with apical protrusion (arrowhead) cytoplasm rich in vacuoles (v), amorphous nucleus (a), and short brush border (bb), Note disorganized regenerative cell nest (rc). L – lumen, n- nucleus. Scale bars: 10 µm

The midgut of control *P. absoluta* larvae presented a single-layered pseudostratified epithelium, with digestive and goblet cells and some regenerative cell nests (Fig. 1A). The digestive cells presented a well-developed apical brush border, an acidophil cytoplasm with some vacuoles, and a spherical nucleus rich in decondensed chromatin (Figs. 1B, C). The goblet cells were characterized by an enlarged apical infolding forming a large extracellular cavity with a well-developed brush border and a basal nucleus with decondensed chromatin (Figs. 1B, C). Furthermore, there are some scattered cells with a granulated basophilic cytoplasm, characterized as intracellular bacteria, forming mycetocytes (Fig. 1B).

Larvae exposed to the LC_50_ of a chlorfenapyr-based insecticide revealed significant midgut damage. Digestive cells exhibited a series of severe alterations, including a short apical brush border, intense cytoplasm vacuolization, apical protrusions, and amorphous nuclei, having an elongated and variable shape (Figs. 1D–F). Furthermore, the nests of regenerative cells were disorganized and altered, and no mycetocytes were observed.

**Fig. 2:**
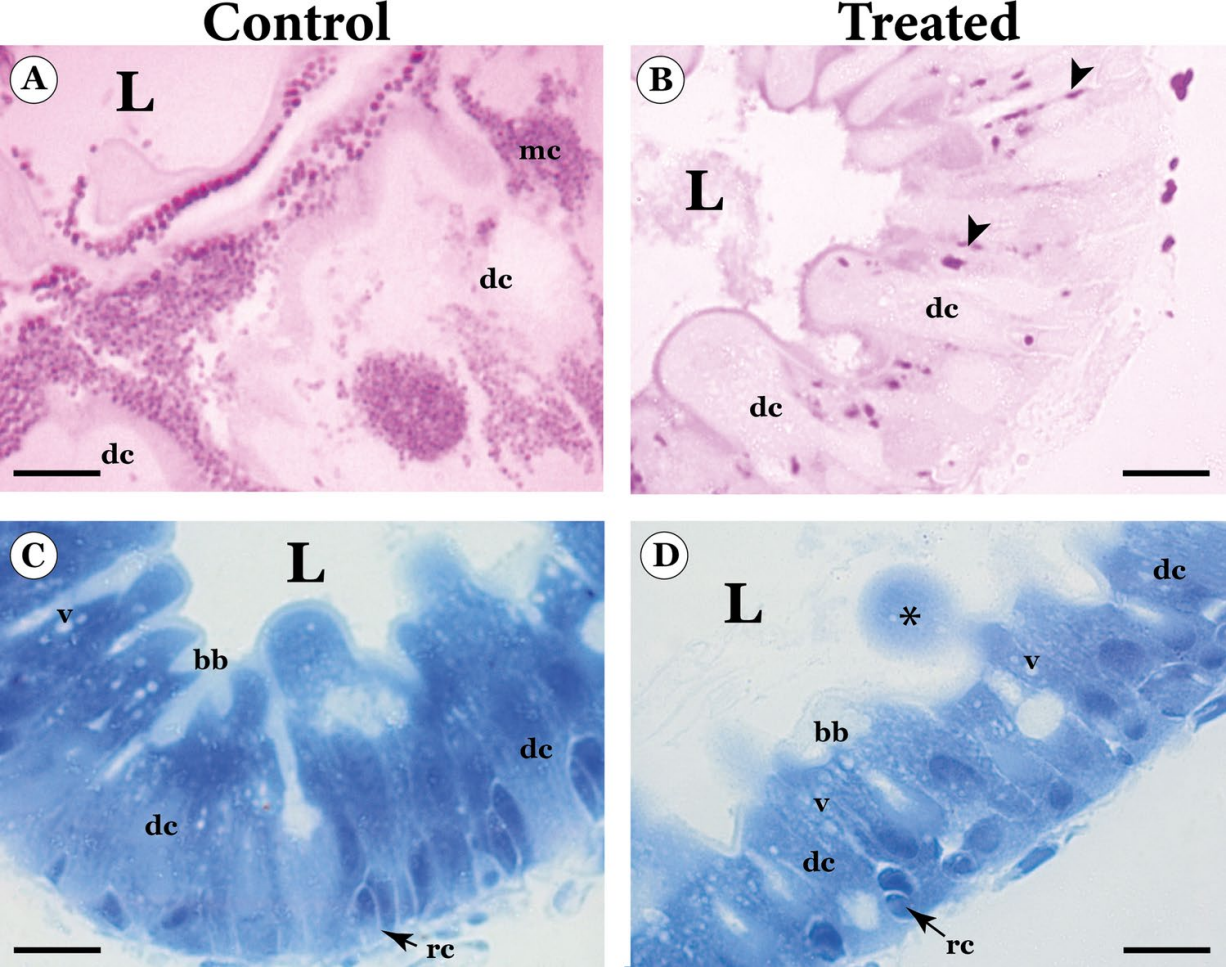
Light micrographs of the histochemical tests in the midgut of the third-instar larvae of *Phthorimaea absoluta* control and orally exposed (treated) to the chlorfenapyr-based insecticide. **A** Midgut epithelium of control larvae showing positive PAS reaction in mycetocytes (mc). **B** Midgut epithelium of treated larvae showing PAS positive reaction in the lateral regions (arrowheads). **C** Midgut epithelium of control larvae showing strong positive reaction for proteins in the apical cytoplasm of digestive cells. **D** Midgut epithelium of treated larvae showing uniform protein positive reaction in the whole cell and in the apical protrusion (asterisk). bb - brush border, dc - digestice cell, L – lumen rc – regenerative cells, v—vacuoles. Scale bars: 10 µm

The P.A.S. histochemical test in the control group revealed a weak reaction in the midgut epithelium, except in the mycetocytes with a strong reaction (Fig. 2A). The midgut of treated larvae showed a strong P.A.S. reaction at the lateral portions of digestive cells and in the brush border, as well as the absence of mycetocytes (Fig. 2B).

The histochemical test for proteins revealed polarity in the midgut epithelium of the control larvae, with strong reaction in the apical cytoplasm and brush border (Fig. 2C). In contrast, chlorfenapyr-based insecticide-treated larvae exhibited uniform positive reaction throughout the midgut epithelium with weaker reactivity in the brush border, and in cell fragments released to the lumen (Fig. 2D).

## Discussion

The chlorfenapyr-based insecticide Pirate® was highly toxic to the evaluated *P. absoluta* population, with an estimated lethal concentration (LC_90_) of 13.8 ppm of active ingredient. This value is approximately ninefold lower than the maximum recommended field dose (120 ppm) and 4.5-fold lower than the minimum field concentration (60 ppm) (MAPA 2022). This discrepancy may result from increased susceptibility in the laboratory population, differences in exposure routes and physiological responses or the extensive use of pesticides in field populations. Field populations of *P. absoluta* from different Brazilian regions exhibit varying levels of susceptibility to insecticides (Silva et al. 2016), although still within limits below recommended field concentrations. Our findings confirm the potential of chlorfenapyr-based insecticide Pirate® in controlling *P. absoluta* populations, consistent with reports for other pesticides such as abamectin and spinosad (Gontijo et al. 2013) and spinetoram and emamectin (Hanafy & El-Sayed 2013).

Histopathological analyses revealed severe damage to the midgut of treated larvae. Chlorfenapyr’s mode of action is primarily associated with mitochondrial dysfunction, a critical process in activating apoptotic cell death pathways (Huang et al. 2013). As expected following acute midgut exposure via ingestion, our results showed digestive cells exhibiting features suggestive of death processes, such as chromatin condensation (amorphous nucleus) and cell fragmentation, similar to those reported in the midgut of *Spodoptera frugiperda* J. E. Smith (Noctuidae) caterpillars exposed to azadirachtin (Huang et al. 2013). In addition to apoptosis, the observed vacuolization of digestive cells in *P. absoluta* larvae exposed to chlorfenapyr is a morphological indicator of cytoplasmic exhaustion and cellular stress, consistent with findings in *S. frugiperda* (Xu et al. 2017), reinforcing the high cytotoxic potential of this insecticide.

Histopathological and histochemical analyses suggested atrophy of the midgut epithelial brush border, a clear indicator of cellular damage that compromises digestive function. The brush border amplifies the apical surface of midgut epithelial cells, playing a critical absorptive and protective role in insects (Serrão & Cruz-Landim 1995). The analyses also suggested alterations in the regenerative cells, which form nests at the base of the digestive epithelium, indicating a deficiency in cellular renewal, thus compromising the insect's recovery (Awais et al. 2024). Damage to the midgut brush border has been widely reported in insects exposed to various xenobiotics (Denecke et al. 2018; Castro et al. 2019).

The absence of mycetocytes in larvae treated with chlorfenapyr-based insecticide may be due directly from the insecticide’s action. Originally developed as an antibacterial compound, chlorfenapyr might alter the selective pressure from the individual insect to its associated microbial community (Siddiqui et al. 2022). Although studies on the role of gut microbiota in Lepidoptera remain limited, these symbionts contribute to xenobiotic detoxification. For instance, gut bacteria in *Plutella xylostella* (Linnaeus) (Plutellidae) are involved in chlorpyrifos detoxification (Xia et al. 2018), while the intestinal symbiont *Enterococcus* has been reported to detoxify chlorantraniliprole in *P. absoluta*, potentially contributing to insecticide resistance (Chen et al. 2024). Considering the essential roles of these symbionts in both digestive activity and detoxification, their absence suggests impaired digestive efficiency and increased insecticide susceptibility in *P. absoluta*.

Histochemical assays for glycoconjugates and protein detection also indicate significant cellular dysfunction and structural disorganization in the midgut of *P. absoluta* larvae treated with chlorfenapyr. The reduced polysaccharide reactivity observed in treated insects may result from insecticide-induced disruption of digestive processes, consistent with findings in *S. frugiperda* exposed to essential oils from *Piper* spp. (Dutra et al. 2019). Furthermore, the abnormal lateral redistribution of carbohydrates in treated larvae would suggest cytoplasmic disorganization and potential cytoskeletal impairment.

Protein histochemistry revealed strong positive reactivity in the apical region of midgut cells in the control group, whereas larvae exposed to the chlorfenapyr-based insecticide Pirate® exhibited a more homogeneous distribution of proteins, likely reflecting compromised digestive activity in the epithelial cells. Additionally, reduced protein abundance was observed in treated larvae, evidenced by a weak positive reaction, suggesting possible damage to cytoskeletal proteins, resulting in pronounced cellular disorganization and extrusion of cellular material (Serra et al. 2023). However, data on *P. absoluta* midgut histopathology are scarce, making this the first study on pesticide-induced structural alterations in this species.

Chlorfenapyr is considered a next-generation pesticide developed to enhance pest management across various crops, particularly in cases where *P. absoluta* populations have developed resistance to compounds such as abamectin and Cartap (Silva et al. 2016). Evidence from this study, in agreement with previous findings (Hanafy & El-Sayed 2013; Silva et al. 2016), supports the high efficacy of the chlorfenapyr-based insecticide against *P. absoluta*. Moreover, its successful application against *S. frugiperda* in maize, combined with the low likelihood of cross-resistance with other insecticides (Kanno et al. 2020), highlights its potential as a valuable tool for chemical control of the tomato leafminer.

In conclusion, our findings demonstrate the toxicity of the chlorfenapyr-based insecticide Pirate® to third-instar larvae of *P. absoluta*, causing significant mortality and extensive histopathological damage to the midgut epithelium, including the loss of mycetocytes. These alterations indicate impaired digestive function and potential induction of anti-feeding behavior. Furthermore, this study provides one of the first histopathological assessments of *P. absoluta* following pesticide exposure, addressing a critical knowledge gap. Given the scarcity of similar investigations in microlepidoptera, our results offer novel insights into the cellular-level impacts of chlorfenapyr and support its continued evaluation as an important tool in integrated pest management programs.

## Data Availability

The datasets used and/or analyzed during the current study are available from the corresponding author on request.
